# Mental Disorders in Individuals With Exercise Addiction—A Cross-Sectional Study

**DOI:** 10.3389/fpsyt.2021.751550

**Published:** 2021-12-09

**Authors:** Maximilian Meyer, Isabel Sattler, Hanna Schilling, Undine E. Lang, André Schmidt, Flora Colledge, Marc Walter

**Affiliations:** ^1^University Psychiatric Clinics, University of Basel, Basel, Switzerland; ^2^Department of Sport, Exercise and Health, University of Basel, Basel, Switzerland

**Keywords:** exercise addiction, SCID-5, exercise dependence, excessive exercising, behavioural addiction, diagnostic screening

## Abstract

**Background and Aims:** Exercise addiction has not yet been designated as an addictive disorder in the DSM-5 due to a lack of detailed research. In particular, associations with other psychiatric diagnoses have received little attention. In this study, individuals with a possible exercise addiction are clinically assessed, in order to establish a profile of co-occurring psychiatric disorders in individuals with exercise addiction.

**Methods:** One hundred and fifty-six individuals who reported exercising more than 10 h a week, and continued to do so despite illness or injury, were recruited for the study. Those who met the cut-off of the Exercise Dependence Scale (*n* = 32) were invited to participate in a screening with the Structured Clinical Interview for DSM-5 (SCID-5-CV) and personality disorders (SCID-5-PD). Additionally, an interview based on the DSM-5 criteria of non-substance-related addictive disorders was conducted to explore the severity of exercise addiction symptoms.

**Results:** 75% of participants fulfilled the criteria for at least one psychiatric disorder. Depressive disorders (56.3%), personality disorders (46.9%) and obsessive-compulsive disorders (31.3%) were the most common disorders. Moreover, there was a significant positive correlation between the number of psychiatric disorders and the severity of exercise addiction (*r* = 0.549, *p* = 0.002).

**Discussion:** The results showed a variety of mental disorders in individuals with exercise addiction and a correlation between the co-occurrence of mental disorders and the severity of exercise addiction. Exercise addiction differs from other addictive und substance use disorders, as obsessive-compulsive (Cluster C), rather than impulsive (Cluster B) personality traits were most commonly identified.

**Conclusions:** Our results underscore the importance of clinical diagnostics, and indicate that treatment options for individuals with exercise addiction are required. However, the natural history and specific challenges of exercise addiction must be studied in more detail.

## Introduction

The term *exercise addiction* can be found in scientific literature dating back to the late 1970s (1). It is used to refer to individuals who lose control over their exercising habits and continue their regimen despite negative physical, psychological, and social consequences (2). Other terms like *obligatory exercise, exercise dependence* and *compulsive exercise* have emerged since (3, 4). Although the symptoms of exercise addiction have been described in the literature for over five decades, robust evidence for the classification as a behavioural disorder is still lacking (5, 6). The literature consists mostly of qualitative data and case reports listing self-reported symptoms of the phenomenon (7–9) while consistent and methodologically solid studies are scarce (2, 10). To assess unhealthy exercising behaviour, scholars have developed questionnaires based on the self-reported symptoms of their study subjects (11). Those questionnaires also highlight that a uniform understanding of the phenomenon is lacking: the Exercise Dependence Scale (EDS) (12) identifies individuals at risk of exercise dependence and the Obligatory Exercise Questionnaire (OEQ) (13) rates whether problems with obligatory exercise can be observed. Colledge et al. (14) have pointed out that in a review of 79 questionnaire-based studies addressing exercise addiction, five different questionnaires are employed, each of which is based on a distinct conceptual framework.

With the introduction of pathological gambling in the fifth edition of the Diagnostic and Statistical Manual of Mental Disorders, “gambling disorder” is still the sole addictive disorder that is found in the category of “non-substance-related disorders.” Other behaviours with possible pathological characteristics have been discussed but not been added due to insufficient peer-reviewed evidence (10). Behaviours that show addictive or pathological traits and are therefore being investigated are internet-related behaviours like gaming (15), as well as sex, shopping, and exercise (5). Notably, there is an ongoing general discussion about the extent to which excessive behaviours can be compared to substance-related addictive disorders (16, 17). A main point of criticism is that criteria for substance-related addictions are often adapted and used for identifying behavioural addictions without it being clear whether those criteria are suitable for pathological behaviours (18). It also bears noting that the guidelines which might constitute a mental disorder are subject of debate (19). The DSM-5 characterises mental disorders as syndromes affecting the individual's cognition, emotion regulation, or behaviour. It further states that significant distress is usually associated with the underlying condition (20). Therefore, the demonstration of psychological distress caused by excessive behaviours is necessary to strengthen the argument for the addition of further non-substance-related disorders. Nonetheless, excessive behaviours show apparent similarities to substance-related addictions: dual-diagnoses are commonly made in individuals suffering from gambling disorder, particularly impulse-control disorders, mood disorders, anxiety disorders and other addictive (substance related) disorders (21). Petry et al. (22) reported that individuals with gambling addiction from a large national sample of the United States also suffered from alcohol use disorder (73.2%), personality disorders (60.8%), affective disorders (49.6%), anxiety disorders (41.3%), and 38.1% had a substance use disorder (excluding alcohol). This is in line with the findings of Kessler et al. (23), who reported that 96.3% of those with gambling disorder also meet the criteria for another mental disorder in their lifetime.

In a recent meta-review Colledge et al. (24) studied the range of symptoms reported in the literature, and proposed criteria for exercise addiction based on the DSM-5 criteria for gambling disorder (Table 1). These criteria are designed to serve as the basis for a clinical screening of symptoms which may indicate exercise addiction. Since these criteria were used in this study to rate the severity of the observed symptoms, the term *exercise addiction* is used concordantly.

**Table 1:** Criteria developed for rating the severity of exercise addiction based on symptoms that have been reported to occur in individuals who exercise excessively (24).

1	Exercise volume has increased over time in order to avoid negative feelings of guilt or laziness
2	Negative affective response when exercise is reduced or sessions are missed/stopped
3	Attempts to reduce exercise volume are feared and/or unsuccessful
4	Is often preoccupied with exercise (e.g., having persistent thoughts of when and where next session will take place, planning training, thinking of ways to exercise during other activities)
5	Exercise is used as a way to cope with negative life experiences or stressors
6	Exercise is continued in spite of illness, injury or severe pain, at levels beyond rehabilitative training
7	Lies about or minimises time and intensity of exercise
8	Has jeopardised or lost a significant relationship, job, or educational or career opportunity because of exercise
9	Despite rational understanding of the negative physical and/or psychological burden of exercise habits, habits are continued
10	Feeling of guilt when exercise is missed or reduced

In case of exercise addiction, the prevalence and range of co-occurring mental disorders has not yet been clinically examined. Eating disorders are frequently discussed within the context of excessive exercising habits (25) and indeed it seems intuitive that exercise might be practised simply for the purpose of weight control. Furthermore, some of the highest prevalence rates of exercise addiction (between 38 and 45%) are reported in individuals with diagnosed eating disorders (26). Exercising behaviour would therefore have to be considered a symptom of the underlying disorder rather than its own entity. In respect to this, the differentiation between *primary* and *secondary exercise dependence* has been proposed, with *primary* meaning that exercising habits are not a *secondary* feature of anorexia nervosa or bulimia nervosa (27). This distinction has been broadly adopted by scholars and the implication has been made that exercise dependence without co-occurring eating disorders is a rare phenomenon (28–30). However, Grandi et al. (31) have explicitly excluded those with eating disorders in their studies, and still found symptoms of exercise addiction, providing evidence that the phenomenon exists apart from eating disorders.

The theory that exercise addiction might be a maladaptive coping-strategy to escape from psychological hardship has also been discussed (4, 32). This is supported by robust evidence that exists for positive effects of physical exercise in individuals with major depression (33, 34). Indeed, it seems plausible that exercise might resemble a self-medicating coping pattern and is being used for affect-regulation and stress relief. It is also possible that exercise addiction might be related to emotional dysregulation (35) and higher levels of alexithymia (36) which are also commonly found in substance use disorders. However, to date, no studies with a longitudinal design examine the chronological relationship between exercise addiction and co-occurring mental disorders to test this theory.

In summary, doubts about the existence of primary exercise addiction and whether it should be regarded as a behavioural addiction are warranted. Although symptoms of exercise addiction are consistently reported in the literature, no clinical studies that examine the prevalence of a variety of mental disorders in individuals with exercise addiction are available. It is therefore unclear whether those with exercise addiction suffer from mental disorders more commonly than the general population, and whether they show the same pattern of co-occurring disorders that has been observed in other behavioural disorders like gambling disorder. It is also unclear whether the severity of exercise addiction symptoms affect and influence co-occurring disorders, and whether those disorders originate from the excessive exercising habits or vice versa.

Thorough clinical diagnostic screening is needed in individuals with exercise addiction to gather data on the prevalence of co-occurring disorders. This would make it possible to investigate whether co-occurring disorders are in fact limited to eating disorders or whether their range matches that of substance use disorders and pathological gambling. If exercise addiction occurs with a varying range of other diagnoses, this could be further evidence that it is a distinct mental disorder. We are the first group worldwide to systematically identify a population of individuals with exercise addiction and conduct gold-standard clinical diagnostic interviews; this study paves the way for a detailed, clinical description of the to-date insufficiently defined phenomenon of exercise addiction.

### Aim

The present study investigates the co-occurrence of mental disorders in individuals with exercise addiction. We hypothesise that the range of co-occurring disorders matches those of other substance-related disorders, such as depressive disorders, personality disorders, and anxiety disorders, and is therefore not limited to eating disorders.

## Materials and Methods

### Participants

Individuals were recruited by advertisements in fitness studios, a local newspaper and via Unimarkt Basel, an online market platform run by the University of Basel. Recruitment took place between 2019 and 2021. The advertisements invited applicants between 18 and 70 years of age, exercising more than 10 h a week, and continuing their exercise despite illness or injuries to participate. These criteria were selected as they were judged to be indicative of a possible addictive disorder in line with DSM-5 criteria, yet also simple and clear enough to be communicated clearly on an advertising flyer or poster. CHF 40 were given for participation in the first examination and an additional 150 CHF were given for further participation in the study.

### Measures

During the initial screening, the Exercise Dependence Scale-21 (EDS-21) was used to identify individuals with possible exercise addiction. The EDS-21 is a validated 21-item questionnaire whose development was based on the DSM-IV criteria for substance use disorders (12). Each criterion (tolerance, withdrawal, intention effect, lack of control, time, reduction in other activities and continuance) is represented by a subscale, consisting of 3 items. Items are rated on a 6-point Likert-scale. Participants who score in the dependent range (i.e., 4 or 5 on the Likert-scale) on at least three criteria are rated “symptomatic, at-risk for exercise dependence” (12). Individuals who met this cut-off were invited to a second examination.

In the second examination, the Structured Assessment of Personality Abbreviated Scale (SAPAS) was conducted. The SAPAS is a brief screening tool for personality disorders that consists of 8 items and showed good sensitivity (0.94) and acceptable specificity (0.85), with the cut-off being set at 3 fulfilled items or more (37). Next, the Structured Clinical Interview for DSM-5 (SCID-5) was conducted to screen for mental disorders. The SCID-5-CV was used in every participant to screen for lifetime prevalence of depressive disorders and currently prevalent Axis I disorders. In addition, the SCID-5-PD was conducted on participants that met the cut-off of the SAPAS score. Third, participants were interviewed about their exercising habits and the extent of their exercise addiction.

Severity of exercise addiction symptomatology was assessed using the 10 item symptom checklist suggested by Colledge et al. (24) and shown in Table 1. These criteria were derived from available literature on the phenomenon of exercise addiction and used to further describe and assess the severity of symptoms. The degree of severity was rated *severe* if 9 or 10 criteria were fulfilled, *moderate* for 7 or 8, *mild* for 5 or 6 or *subclinical* if the participant fulfilled 4 criteria or less.

### Procedure

The EDS-21 was provided in paper-pencil form. Interviews were held face-to-face. Following the outbreak of COVID-19, the EDS-21 was provided online and interviews were held via the conferencing platform *Zoom*.

### Statistical Analysis

Data analysis was conducted using SPSS version 27. A one-way ANOVA was conducted to find out whether there was a difference between exercise addiction severity groups (subclinical, mild, moderate, and severe) and mean number of mental disorders in each group. A *post-hoc* Tukey test was used to identify which groups differ from each other. Level of significance was set at *p* < 0.05.

Pearson's r correlation coefficient was calculated to find out whether the number of fulfilled exercise addiction criteria correlated with total number of mental disorders. Two-sided level of significance was set at *p* < 0.05.

An independent *t*-test was conducted to find out whether the number of fulfilled exercise addiction criteria differed in individuals who participated in athletic competitions. Two-sided level of significance was set at *p* < 0.05.

Outliers exceeding two standard deviations in number of disorders were excluded for the calculation of Pearson's r and one-way ANOVA (*n* = 2).

## Results

### Sample Description

One hundred and fifty-six individuals were recruited for screening, of which 32 met the cut-off of the EDS-21 and were invited to the second examination. Mean age of our sample was 27.9 years (SD = 12.5), comprising 16 men and 16 women. Exercise hours per week ranged from 5 to 55 (M = 17.2, SD = 10.3). Although a minimum of 10 h exercise per week was an explicit inclusion criterion, it emerged that one participant reduced their exercise activity between recruitment and completion of the interview, accounting for this discrepancy. 46.9% (*n* = 15) reported participation in competitions. The level of competition ranged from local leagues to national and world championship participation. None of our participants was a full-time professional athlete. Most participants reported doing multiple different sports. Fitness (strength training) was most common (*n* = 23), followed by running (*n* = 14) and cycling (*n* = 12). Other disciplines varied greatly and included handball, CrossFit, tennis, hiking, martial arts, yoga, basketball, hammer throw, swimming and climbing, among others.

### Descriptive Statistics

Exercise addiction severity ranged from subclinical (0–4 criteria) to severe (9–10 criteria). A total of 8 (25%) participants fulfilled 4 criteria or fewer. Mild exercise addiction (5–6 criteria) was found in 25% (*n* = 8), moderate exercise addiction (7–8 criteria) was found in 37.5% (*n* = 12) and severe exercise addiction (9–10 criteria) was found in 12.5% (*n* = 4).

Participants were diagnosed with 32 different mental disorders as defined by the DSM-5, with a total of 100 diagnoses. Major depressive disorder was the most common and was found in 46.9% (*n* = 15), followed by obsessive-compulsive personality disorder (*n* = 8, 25%) and insomnia (*n* = 8, 25%). The complete range of disorders is listed in Table 2. No mental disorder was found in 25% (*n* = 8). One participant with moderate exercise addiction was found to have no mental disorders. All participants with more than seven fulfilled exercise addiction criteria also met the diagnostic criteria for at least one mental disorder.

**Table 2:** DSM-5 chapters and their according disorders currently prevalent in our sample (*n* = 32).

**DSM-5 chapter**			**Disorder**		
	***N^1^***	**% in sample**		***N^2^***	**% in sample**
Depressive disorders	18	56.3	Major depressive disorder	15	46.9
			Premenstrual dysphoric disorder	7	21.9
Personality disorders	15	46.9	Obsessive-compulsive personality disorder	8	25
			Avoidant personality disorder	5	15.6
			Narcissistic personality disorder	3	9.4
			Schizoid personality disorder	2	6.3
			Antisocial personality disorder	1	3.1
			Borderline personality disorder	1	3.1
			Histrionic personality disorder	1	3.1
Obsessive-compulsive and related disorders	10	31.3	Body dysmorphic disorder	5	15.6
			Hoarding disorder	4	12.5
			Excoriation disorder	4	12.5
			Obsessive-compulsive disorder	1	3.1
			Trichotillomania	1	3.1
Anxiety disorders	9	28.1	Social anxiety disorder	6	18.8
			Generalised anxiety disorder	2	6.3
			Specific phobia (Animal type)	2	6.3
			Panic disorder	1	3.1
Sleep-wake disorders	9	28.1	Insomnia disorder	8	25
			Hypersomnolence disorder	2	6.3
Neurodevelopmental disorders	6	18.8	Attention-deficit/hyperactivity disorder	6	18.8
Substance-related and addictive disorders	5	15.6	Alcohol use disorder	3	9.4
			Gambling disorder	1	3.1
			Stimulant use disorder	1	3.1
			Cannabis use disorder	1	3.1
			Other (Anabolic Steroids) substance–related disorders	1	3.1
Feeding and eating disorders	5	15.6	Anorexia nervosa	3	9.4
			Bulimia nervosa	1	3.1
			Avoidant/restrictive food intake disorder	1	3.1
Disruptive, impulse-control, and conduct disorders	1	3.1	Intermittent explosive disorder	1	3.1
			(Antisocial personality disorder)	(1)	(3.1)
Trauma- and stressor-related disorders	1	3.1	Posttraumatic stress disorder	1	3.1
Bipolar and related disorders	1	3.1	Bipolar I disorder	1	3.1
			Total	100	

*Lifetime prevalence is given for Major Depressive Disorder, including recurrent and single episode. Antisocial personality disorder is found in two chapters in the DSM-5 which was accounted for in our total number of diagnosed disorders. N^1^ = number of subjects who fulfilled the criteria for one or more disorders from the respective DSM chapter. N^2^ = number of subjects who fulfilled the criteria for the specific disorder*.

**Figure 1:**
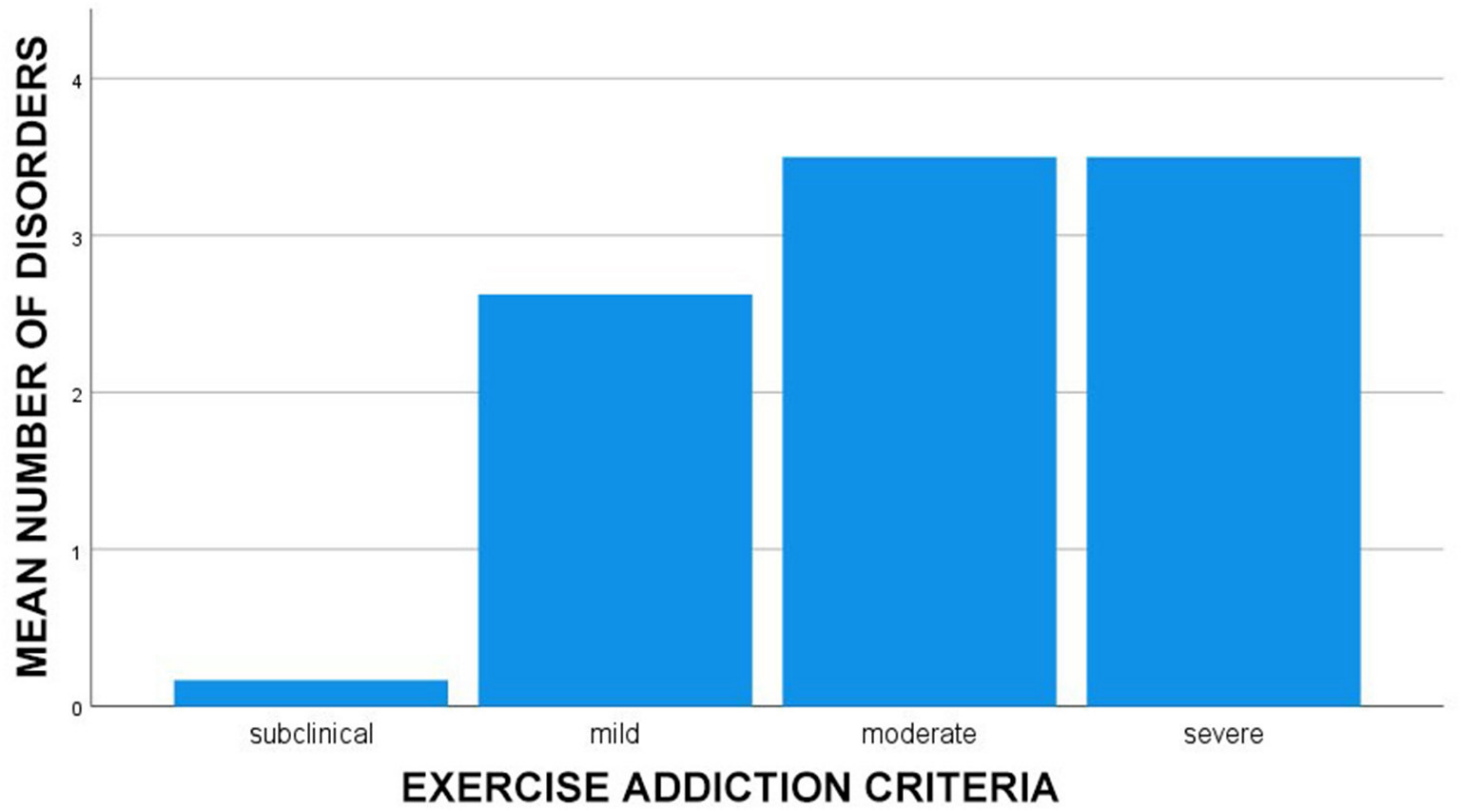
Mean number of mental disorders by severity of exercise addiction (*n* = 30). Outliers exceeding two standard variations in number of disorders (*n* = 2) were excluded.

There was a statistically significant difference between exercise addiction severity groups on total number of mental disorders as determined by one-way ANOVA [*F*_(3, 26)_ = 3.54, *p* = 0.028]. The total number of disorders increased from subclinical exercise addiction (M = 0.17, SD = 0.41), to mild exercise addiction (M = 2.63, SD = 2.72), to moderate exercise addiction (M = 3.50, SD = 2.89), to severe exercise addiction (M = 3.50, SD = 2.40) (see Figure 1). A *post-hoc* Tukey test showed that only the subclinical group and the moderate group differed significantly at *p* < 0.05 (*p* = 0.021).

**Figure 2:**
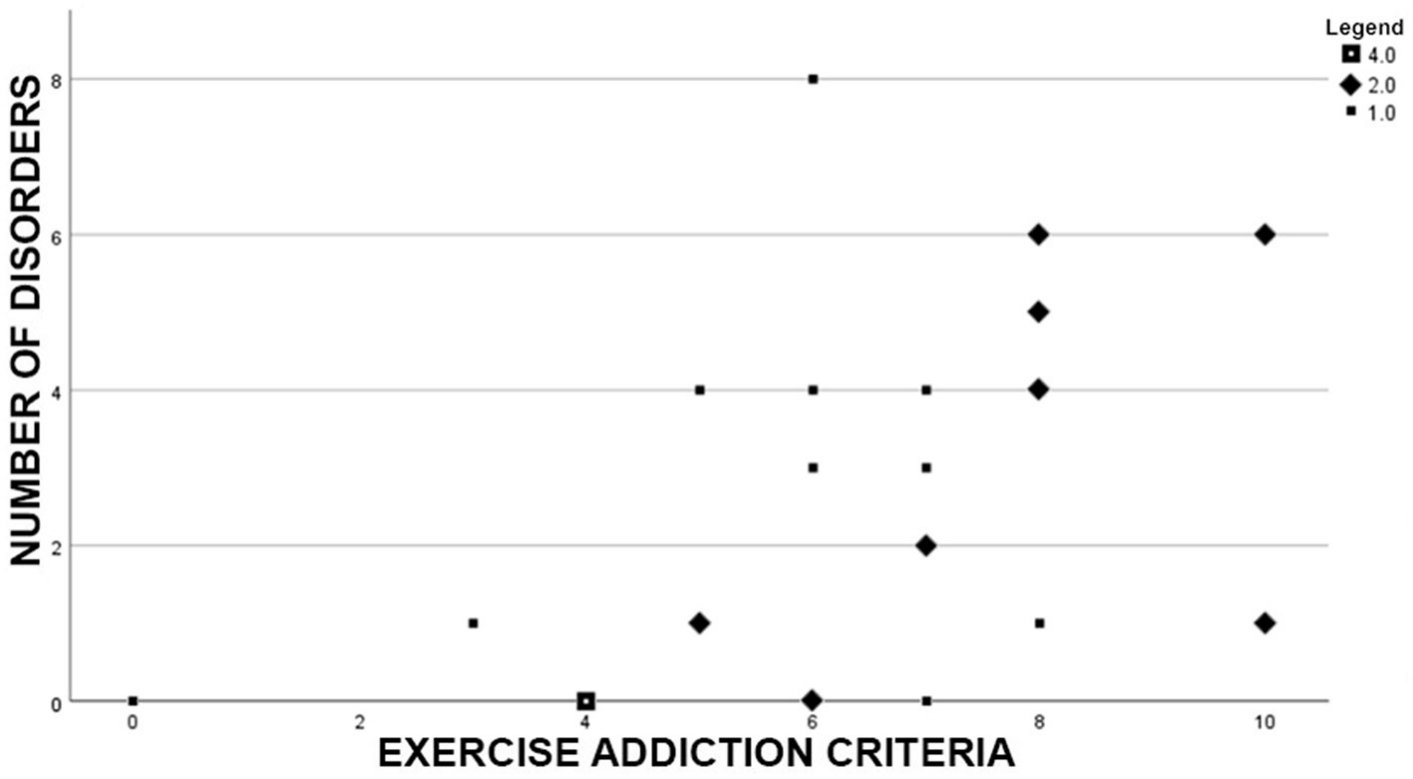
Scatterplot of our sample (*n* = 30) demonstrating the correlation between number of fulfilled exercise addiction criteria and number of disorders. Outliers exceeding two standard variations in number of disorders (*n* = 2) were excluded.

The total number of disorders showed a strong positive correlation to the severity of exercise addiction (*r* = 0.506, *p* = 0.004) and is demonstrated in Figure 2.

Table 3 compares found disorders according to their DSM-5 chapter in competing and non-competing participants. There was no statistically significant difference in the number of fulfilled exercise addiction criteria [t_(30)_ = −0.20, *p* = 0.369] of participants who competed (M = 6.40, SD = 2.72), when compared to the rest of the sample (M = 6.24, SD = 2.05).

**Table 3:** Comparison of mental disorders by DSM-5 chapter between competing and non-competing participants.

	**Competing sample**		**Non-competing**	
	**(*****n*** **=** **15)**		**sample (*****n*** **=** **17)**	
**DSM-5 chapter**	***N^1^***	**% in sample**	***N^1^***	**% in sample**
Depressive disorders	9	60.0	9	52.9
Personality disorders	6	40.0	9	52.9
Obsessive-compulsive and related disorders	7	46.7	3	17.6
Anxiety disorders	4	26.7	5	29.4
Sleep-wake disorders	5	33.3	4	23.5
Neurodevelopmental disorders	4	26.7	2	11.8
Substance-related and addictive disorders	2	13.3	3	17.6
Feeding and eating disorders	2	13.3	3	17.6
Disruptive, impulse-control, and conduct disorders	1	6.7	-	-
Trauma- and stressor-related disorders	-	-	1	5.9
Bipolar and related disorders	-	-	1	5.9

*N^1^ = number of subjects who fulfilled the criteria for one or more disorders from the respective DSM chapter*.

## Discussion

This is the first study to systematically identify individuals with exercise addiction and conduct a gold-standard psychiatric diagnostic screening. Only individuals that met the cut-off of the EDS-21 (12) were invited for diagnostic screening. We found a high prevalence of mental disorders in individuals with exercise addiction, with 75% suffering from at least one disorder listed in the DSM-5. Additionally, the spectrum of disorders we found was broad: 32 different disorders were diagnosed in a sample of 32 participants (see Table 2).

The most common comorbidities in gambling disorder have been found to be alcohol use disorder, personality disorders, affective disorders, and anxiety disorders (22). Cluster B personality disorders are found the most in gambling addiction, followed by Cluster C and Cluster A disorders (38). In our sample, depressive disorders (56.3%), personality disorders (46.9%) and obsessive-compulsive disorders (31.3%) were the most common (Table 2). Cluster C personality disorders were particularly common with 40.6% of participants suffering from either obsessive-compulsive personality disorder (25%) or avoidant personality disorder (15.6%). This finding suggests that impulsive personality traits might be less prevalent in individuals with exercise addiction who rather seem to be located at the compulsive pole of the diagnostic spectrum. Notably, scholars have previously discussed two different dimensions of “unhealthy exercise”: a quantitative dimension (“excessive”) that takes duration, frequency and intensity of exercise into account and a qualitative dimension (“compulsive”), which is characterised by rigid exercise schedules, prioritisation of exercise over other activities and feeling of guilt and anxiety when exercise sessions are missed (39). As addiction is defined in the DSM-5 in terms of cognitive, physical, and behavioural characteristics, and not in quantitative terms, the “compulsive” dimension appears to be more relevant in the identification of exercise addiction.

The total number of mental disorders in our sample increased with exercise addiction severity. Additionally, there was only one participant without a mental disorder who fulfilled seven or more exercise addiction criteria. While our statistical analysis does not account for the clinical severity of the respective conditions, this finding implies that with an increasing number of exercise addiction criteria the occurrence of a mental disorder is also more likely. This indicates that exercise addiction is found in individuals who are more likely to require psychiatric treatment.

Most interestingly, only five participants in our sample suffered from an eating disorder. This contradicts previous studies which reported a strong link between exercise addiction and eating disorders (28–30). The variety of sport disciplines practised in our sample supports the findings of Lichtenstein et al. (40), who found no difference in the prevalence of exercise addiction between team sports and endurance sports.

Besides eating disorders, exercise addiction might be secondary to other mental disorders such as obsessive-compulsive disorders. Scholars have previously reported on primary exercise addiction to show traditional addictive traits, whereas secondary exercise addiction was found to be more compulsive in nature (30). No strong link between exercise addiction and eating disorders was observed in our sample. However, this is not the case for obsessive-compulsive disorders and obsessive-compulsive personality disorders. Therefore, we are not able to rule out that exercise addiction symptoms might be secondary to this part of the diagnostic spectrum. Literature also indicates an association between body dysmorphic disorder and exercise addiction symptoms (41). However, our data suggests that these symptoms are not limited to body dysmorphic disorder or obsessive-compulsive disorder alone, as we found that only five and one participant(s) respectively met the diagnostic criteria for each specific disorder.

It might also be possible that our sample included multiple heterogenous groups of aspiring athletes, individuals suffering from eating disorders and individuals with body dysmorphic disorder, all of which would require a more careful interpretation of the respective role of exercise addiction symptoms. However, we do not think that this was the case regarding participation in athletic competitions. Even though we did not conduct statistical inference, the descriptive comparison of disorders by competing and non-competing participants (Table 3) did not differ noticeably. We also did not find a statistically significant difference between competing and non-competing participants in number of fulfilled exercise addiction criteria.

More than half of our sample suffered from a depressive disorder (56.3%) in their lifetime, with major depressive disorder being the most common (46.9%). Since exercise has been found to have positive effects on depression (33, 34), the theory that excessive exercise is used as self-medicating coping-pattern seems plausible. Further studies are needed to investigate the natural history of exercise addiction and its connexion to the emergence of depression longitudinally.

The importance of clinical distress caused by conditions that qualify as mental disorders is emphasised by the DSM-5 and in scientific literature (19). It bears noting, that we did not assess clinical distress or disability in detail or by the means of validated instruments specifically developed for this purpose. The 10 criteria for exercise addiction (Table 1) as well the fact that all participants scored above the threshold of the EDS-21 served as our measure for the presence of significant distress, as both instruments formulate items based on the experience of discomfort, distress, and impairment.

Finally, it is important to note that despite meeting and exceeding the EDS-21 cut-off for exercise dependence, we found that in 25% of our sample <5 exercise addiction criteria applied. This is a further indication that self-report questionnaires addressing exercise addiction may need to be redesigned, as numerous (2, 42, 43) studies now suggest that they are not adequate in differentiating between sportspeople and individuals with an addictive disorder.

## Conclusion

Depression, Cluster-C personality disorders and other obsessive-compulsive disorders appear to be particularly common in subjects with exercise addiction. Our data therefore provides an indicator that the phenomenon of exercise addiction does co-occur with a variety of mental disorders. Our study shows that the phenomenon of exercise addiction occurs independently of eating disorders and body dysmorphic disorders. Since this is a cross-sectional study, it remains unclear whether symptoms of exercise addiction remain stable over time (and in relation to the respective mental disorders). It is also entirely possible that the observed exercising behaviour resembles a coping-strategy to deal with other psychological conditions or current life circumstances not detected in our interview. To deal with this limitation, exercise addiction symptoms need the be observed over longer periods of time to gather longitudinal data and to investigate the requirement of psychiatric and psychotherapeutic treatment.

## Limitations

Our study has several limitations. First, our sample size was small, which does not allow us to study the potential secondary nature of exercise addiction in different subgroups in more detail. Second, we used a cross-sectional design and are therefore not able to draw conclusions about the stability of symptoms over time. Third, we did not sample a matched control group (e.g., individuals who exercise more than 10 h a week but did not meet the EDS-21 cut-off) and are therefore not able to establish, whether the total number of mental disorders we found is in fact excessive among the population we studied. Finally, we used exercise addiction criteria as proposed in literature to assess exercise addiction severity. However, this scale is not a validated instrument, and its psychometric properties are unknown.

## Data Availability Statement

The raw data supporting the conclusions of this article will be made available by the authors, without undue reservation.

## Ethics Statement

The study procedures were carried out in accordance with the Declaration of Helsinki. The study was approved by the *Ethikkommission Nordwest- und Zentralschweiz* (EKNZ) on May 10th, 2019. All subjects were informed about the study and all provided informed consent.

## Author Contributions

MM wrote the first draft, conducted clinical interviews, performed statistical analysis, and takes responsibility for the integrity of the data and the accuracy of the data analysis. IS conducted clinical interviews. HS recruited participants and conducted initial screening. FC obtained funding. MW supervised the study. FC and MW designed the study and wrote the protocol. UL and AS revised the manuscript and provided substantial intellectual input. MM, IS, and FC had full access to all data in the study. All authors contributed to and have approved the final manuscript.

## Funding

Funding for this study was provided by the Gertrud Thalmann Fonds of the University Psychiatric Clinics (UPK) Basel. The funding source was not involved in the study design, collection, analysis or interpretation of the data, writing the manuscript, or the decision to submit the paper for publication.

## Conflict of Interest

The authors declare that the research was conducted in the absence of any commercial or financial relationships that could be construed as a potential conflict of interest.

## Publisher's Note

All claims expressed in this article are solely those of the authors and do not necessarily represent those of their affiliated organizations, or those of the publisher, the editors and the reviewers. Any product that may be evaluated in this article, or claim that may be made by its manufacturer, is not guaranteed or endorsed by the publisher.
